# A randomized controlled trial for effectiveness of zolpidem versus acupressure on sleep in hemodialysis patients having chronic kidney disease–associated pruritus

**DOI:** 10.1097/MD.0000000000010764

**Published:** 2018-08-03

**Authors:** Inayat Ur Rehman, David Wu Bin Chia, Raheel Ahmed, Nisar Ahmad Khan, Aziz Ur Rahman, Syed Munib, Learn Han Lee, Kok Gan Chan, Tahir Mehmood Khan

**Affiliations:** a School of Pharmacy, Monash University, Jalan Lagoon Selatan, Selangor Darul Ehsan, Malaysia; b Department of Pharmacy, Abdul Wali Khan University, Mardan; c Department of Nephrology, Institute of Kidney Diseases; d Department of Nephrology, North West General Hospital and Research Center, Peshawar, Pakistan; e Department of Urology, North West General Hospital and Research Center, Peshawar, Pakistan; f Biomedical Research Laboratory, Jeffrey Cheah School of Medicine and Health Sciences, Monash University Malaysia, 47500 Bandar Sunway, Selangor Darul Ehsan, Malaysia; g Center of Health Outcomes Research and Therapeutic Safety (Cohorts), School of Pharmaceutical Sciences, University of Phayao, Phayao, Thailand; h International Genome Centre, Jiangsu University, Zhenjiang, China; i ISB (Genetics), Faculty of Science, University of Malaya, 50603 Kuala Lumpur, Malaysia; j Institute of Pharmaceutical Sciences, University of Veterinary and Animal Science, Outfall Campus, Civil Lines, Lahore, Pakistan.

**Keywords:** acupressure, chronic kidney disease–associated pruritus, hemodialysis, Pakistan, quality of life, sleep quality, zolpidem

## Abstract

**Background:**

Pruritus adds to the complications of chronic kidney disease (CKD) patient and a well-recognized complication among the CKD patients. Majority of the patients on hemodialysis experience a generalized pruritus and patients reported being moderately to extremely disturbed by at least one of the sleep-related condition. This study aim to investigate the effectiveness of zolpidem 10 mg and acupressure therapy on foot acupoints to improve the sleep quality and overall quality of life among hemodialysis patients suffering from CKD-associated pruritus.

**Methods:**

A multicentered, open-label, parallel group, prospective randomized controlled trial among patients suffering from CKD-associated pruritus with sleep disturbance, after randomization into control, and intervention group to be held at North West General Hospital and Research Center Peshawar, Pakistan and Institute of Kidney Diseases Peshawar, Pakistan.

**Results:**

The primary outcome is to investigate the effectiveness of zolpidem 10 mg and acupressure therapy on foot acupoints to improve the sleep quality and overall quality of life among hemodialysis patients suffering from CKD-associated pruritus. After baseline assessment by Urdu version of 5D itch scale and Urdu version of Pittsburgh Sleep Quality Index (PSQI) and Urdu EQ-5D 3L, the intervention group will be given zolpidem 10 mg oral tablets and control group with acupressure on both foots on KI-1 acupoints for total of 6 minutes. Assessment will be done at weeks 4 and 8 from baseline by using Urdu version of 5D itch scale and Urdu version of PSQI and Urdu EQ-5D 3L, whereas safety profiling of zolpidem 10 mg tablet at week 6 from baseline and acupressure acceptability at week 6 from baseline. Analysis of covariance will be used to examine the differences in treatment effects between the intervention and control groups.

**Conclusion:**

Improvement of sleep quality and quality of life among patients with CKD-associated pruritus requires great importance. This study aims to improve the quality of sleep and quality of life among patients with hemodialysis suffering from CKD-associated pruritus.

## 1 Introduction

Chronic kidney disease (CKD) specially the last stages of CKD [end-stage renal disease (ESRD)] emerged as life-threatening problem globally.^[1,2]^ Overall, during the last 10 years the CKD-associated mortality has increased by 31.7%.^[3]^ Previously the incidence of pruritus among patients with CKD was very high up to 90%^[4]^; however, due to better understanding of pruritus the prevalence dropped down to 60% to 70% in mid of 1980.^[5,6]^ Pruritus adds to the complications of patient with CKD and a well-recognized complication among the them.^[5,7,8]^ CKD-associated pruritus has been reported from 22% to 84% in different studies.^[4,9,10]^ Majority of the patients on hemodialysis experience a generalized pruritus; however, the pruritus can be localized to the chest, limb, back, or head and it can be intermittent or prolonged for hours and days that usually worsen at night.^[11,12]^ Sleep problem is very common among patients suffering from CKD-associated pruritus undergoing dialysis. From previous studies it is evident that patients suffering from CKD-associated pruritus possess lower health-related quality of life including poor sleep quality.^[13–16]^ Similarly; a study by Wikstrom B reported pruritus by 75%; approximately 72% of patients reported being moderately to extremely disturbed by at least 1 of the sleep-related condition,^[17]^ whereas Einollahi et al^[18]^ reported 60.6% of hemodialysis patients had experienced poor sleep resulted from pruritus. Overall, the sleep disorder due to CKD-associated pruritus ranges from 28% to 90%.^[14,18–21]^ Poor sleep quality is linked to increased risk of hypertension,^[22]^ CKD progression,^[23,24]^ elevated mortality rate,^[25]^ and higher healthcare utilization.^[26]^

In Pakistan it is reported that every third person is suffering from kidney disease;^[27]^ patients with ESRD are constantly rising with estimated annual incidence of 100 per million populations.^[28]^ The prevalence of CKD-associated pruritus among hemodialysis patients ranges from 64% to 77.7%.^[12,29–32]^ Difficulty in falling to sleep due to pruritus was reported in 55% of patients, whereas awakened by pruritus was reported in 13% of patients undergoing hemodialysis in a study done in Pakistan.^[12]^

Currently the therapeutic interventions supposed to improve sleep quality in dialysis patients include pharmacotherapy with hypnotic agents,^[33]^ pharmacotherapy with wide range of therapeutic agents for treatment and relief of uremic pruritus^[34]^ ultimately improvement in sleep; cognitive behavioral therapy,^[35]^ for example, sleep hygiene^[36]^ and relaxation.^[37]^ Among the pharmacotherapy with hypnotic agents for insomnia among patients with CKD, nonbenzodiazepine hypnotics are considered to be an alternative hypnotic agents in dialysis centers because of no physical dependence, good effects, no active metabolites, and no or least adverse effects regarding inducing sleep apnea.^[38–40]^ In the study by Dashti-Khavidaki et al^[41]^ among hemodialysis patients by using zolpidem versus clonazepam, zolpidem was not associated with undesirable sleep side effects such as daytime drowsiness, headache, or amnesia. Furthermore, zolpidem was well tolerated in hemodialysis patients, patients did not complain of any particular side effects and zolpidem significantly improved the sleep quality.^[41]^ Among nonpharmacological interventions, acupressure is applied at specific meridians or acupoints in Traditional Chinese Medicine to improve sleep quality. Unlike pharmacological and other interventions acupressure is a noninvasive therapy, which is likely to be associated with a low risk of side effect profile.^[42]^

Keeping in view the importance impaired quality of life due to CKD-associated pruritus patients on hemodialysis, it is vital to do a randomized controlled trial (RCT) to improve the sleep quality of these patients. As no intervention study has been done in Pakistan to improve sleep quality of CKD-associated pruritus patients on hemodialysis. This intervention study is the first of its kind to focus on safety of intervention, that is, zolpidem and acupressure and to establish improvement of sleep quality by reduction in Pittsburgh Sleep Quality Index (PSQI) score among patients suffering from CKD-associated pruritus undergoing hemodialysis. We designed 8-month intervention model to improve sleep quality among CKD-associated pruritus patients on hemodialysis in Pakistan.

## 2 Study aims

### 2.1 Primary aim

To investigate the effectiveness of zolpidem 10 mg and acupressure therapy on foot acupoints to improve the sleep quality and overall quality of life among hemodialysis patients suffering from CKD-associated pruritus.

#### 2.1.1 Secondary aim 1

To establish the safety of zolpidem 10 mg among hemodialysis patients suffering from CKD-associated pruritus.

#### 2.1.2 Other aims

Patient acceptability toward acupressure by “Treatment Acceptability Questionnaire (TAQ).”

## 3 Method

### 3.1 Study design

**Figure 1:**
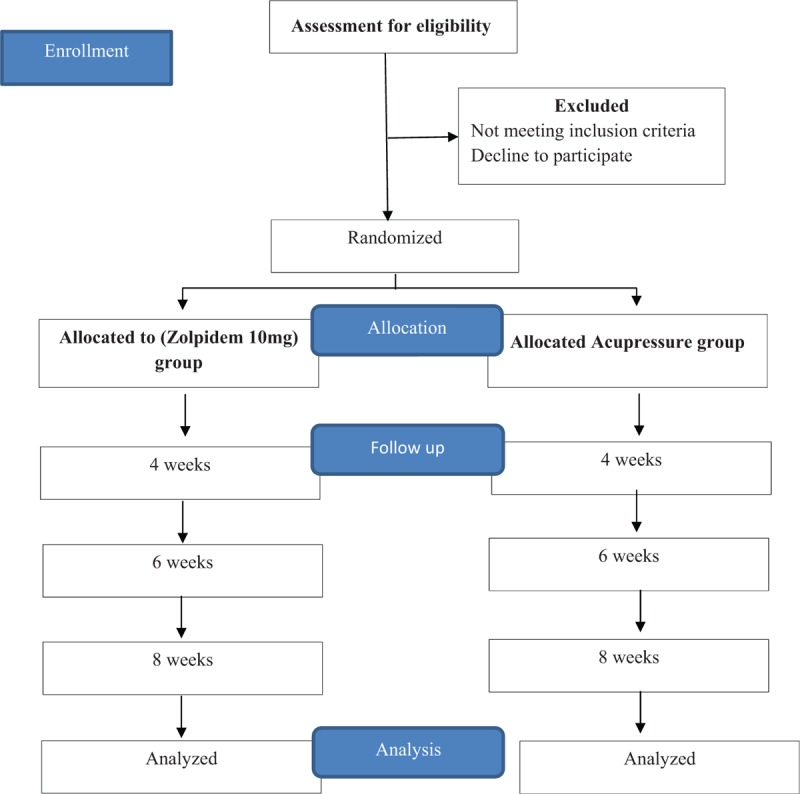
Study flow diagrams.

This is a multi-centered, open-label, parallel group, prospective RCT. In this study effectiveness and safety of zolpidem 10 mg and acupressure therapy on foot acupoints and patient acceptability toward acupressure will be investigated among hemodialysis patients suffering from CKD-associated pruritus. The study purpose will be explained, and patients’ consent will be obtained before enrolling by the principle investigator. The study flow is presented in Figure 1.

### 3.2 Study setting

This trial would be conducted from March 2018 to June 2018 at North West General Hospital and Institute of Kidney Diseases Peshawar, Pakistan.

### 3.3 Ethics and dissemination

The study protocol was approved by the Ethical and Research Committee of North West General Hospital and Institute of Kidney Diseases Peshawar, Pakistan, and will be approved from Monash University Human Research Ethics Committee, and the trial is registered under Australia New Zealand Clinical Trial Registry Trial ID: ACTRN12618000001291, on 8/01/2018. All procedures performed in this study involving human participants are in accordance with the ethical standards of the institutional and/or national research committee and with the 1964 Helsinki declaration and its later amendments or comparable ethical standards. Informed written consent will be obtained from all the study participants, after explaining them the purpose and procedure of the study. Participants can withdraw from the study willingly at any point of the study. Proper patient identity numbers will be given to each patient to use for future reference throughout the study. All data will be highly confidential to minimize any bias.

### 3.4 Study population

Eligibility criteria for patients include adult patients (above age of 18 years), diagnosed with end-stage kidney disease having CKD-associated pruritus affecting sleep quality; not on any sleep medicines and on any treatment to treat pruritus, all the patients must be receiving hemodialysis twice or thrice weekly. Participants will be excluded if patients were not having CKD-associated pruritus, PSQI score <5, no willing to participate and on any medication for sleep and pruritus management.

### 3.5 Study procedure

This trial has 2 phases,

**Phase 1:** (recruitment and screening of patients)

Patients who will meet the inclusion criteria of the study will be recruited for participation in the study. If the patient is willing to participate in the study, a written informed consent for the participation in the study will be obtained and baseline assessment will be done. The participant's pruritus score will be determined on Urdu 5D itch scale and sleep score on Urdu version of PSQI and quality of life impaired due to poor sleep by Euro quality of life 5D 3L (EQ5D 3L) Urdu version.

**Phase 2:** (intervention)

To ensure adequate concealment of allocation, the patients handpick a numbered envelope from the basket. After recruitment, the patients will be requested to handpick an envelope from the basket indicating allocation to groups.

Participants will be randomized 1:1 into 2 group in which 1 group patients will receive zolpidem (oral tablet) 10 mg once daily and other group will receive acupressure therapy (control group) on acupressure points KI-1 points in both foots daily. The therapy will be applied at acupoints KI-1 (Yongquan) in total 6 minutes with 3 minutes per foot and the applied intensity will be adjusted per patient's level of tolerance for 8 weeks at North west General Hospital and Research Center Peshawar Pakistan and Institute of Kidney diseases Peshawar.

At week 4 and week 8 from the baseline the participants in both groups control and intervention group will be required to fill the PSQI questionnaire to assess improvement in sleep quality and improvement in PSQI score and improvement in quality of life impaired due to poor sleep by EQ5D 3L Urdu version. Naranjo algorithm known to be a valid measure for reporting and authenticating drug-related events will be required to fill. All patients completing 6 weeks of zolpidem 10 mg (orally) once daily therapy will be questioned about any adverse events that they may have experienced after taking zolpidem (oral tablet) 10 mg once daily. The information will be collected based on the patients experience and the list of the adverse events. At week 6 the patients will be questioned about acceptability of acupressure therapy by using TAQ. The details of study schedule for data collection are presented in Table 1.

**Table 1:**
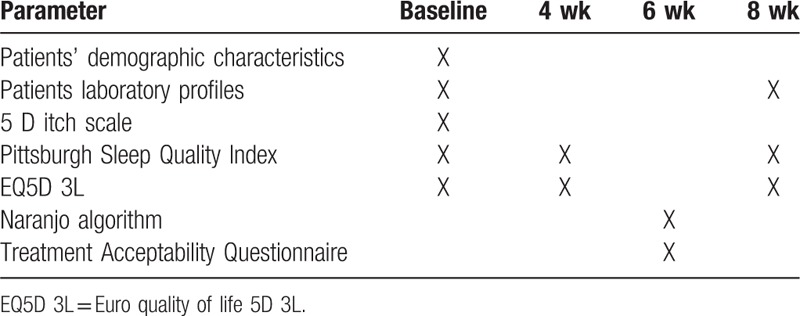
Study schedule for data collection for intervention and control groups.

### 3.6 Measurement tools

#### 3.6.1 Urdu 5D-itch scale

Urdu version of 5D-itch scale^[32]^ will be used for assessment of pruritus among participants. The 5D-itch scale comprises of 5 domains: duration, degree, direction, disability, and distribution.^[43]^ Domains such as duration, degree, and direction involve 1 item, whereas the disability domain contains 4 items, that is, sleep, leisure/social activities, housework, and work/school, each on a 5-point Likert scale. The score of duration, degree, and direction domains are on scale 1 to 5 while for the disability domain the highest score of among any of the 4 items to be taken. The last domain of 5D-IS is distribution; 16 body parts are included in this domain and the participants select which part of their body is affected by pruritus. Five scoring bins were constructed for distribution domain; range of 0 to 2 was categorized as 1, 3 to 5 was categorized as 2, 6 to 10 was categorized as 3, 11 to 13 was categorized as 4, and 14 to 16 was categorized as 5. The score of 5D-itch scale ranges between 5 for no pruritus and 25 for severe pruritus.^[43]^

#### 3.6.2 Pittsburgh Sleep Quality Index

PSQI will be used of assessing sleep quality affected by pruritus among patients with CKD based on the previous studies. The PSQI was designed by Buysse et al^[44]^ to assess sleep quality during past month. It contains in which 7 “component” scores include subjective sleep quality, sleep latency, sleep duration, habitual sleep efficiency, sleep disturbances, use of sleeping medication, and day time dysfunction. PSQI questions 1 to 4 contain information related to bed time and length of time to fall asleep. Questions 5 to 8 are answered on a 0 to 3 scale with 0 indicating no symptom presence and 3 representing symptoms present 3 or more times the past week. Question 9 is answered on a 0 to 3 scale with 0 meaning “very good” and 3 representing “very bad.” The 7 component scores are summed to give 1 global score.^[44]^ All scores are to be combined according to the scoring criteria included with the form to produce a Global PSQI Score. The scores >5 indicate disturbed or poor sleep.^[44]^

#### 3.6.3 Euro quality of life 5D 3L

The EQ5D-3L comprises 5 dimensions related to quality of life: mobility, self-care, usual activities, pain/discomfort, and anxiety/depression. Each dimension has 3 levels with responses to be recorded on no problems, some problems, and extreme problems.^[45]^ The EQ5D-3L also includes a visual analogue scale that records the respondent's self-rated health status on a graduated scale ranging from 0 to 100, in which 100 score indicate good quality of life, whereas score of 0 represents worst quality of life. The Urdu version of EQ5D-3L was provided by EuroQol upon request.

#### 3.6.4 Naranjo algorithm

Naranjo algorithm known to be a valid measure for reporting and authenticating drug-related events.^[46,47]^ Naranjo algorithm is a 10-item scale with 3 options (“yes,” “no,” and “don’t know”) to express the occurrence of a drug-related incident. Based on these 3 options, a score is assigned for each item. If the total score is of >9, it reflects that the AEs are due to the drug being used by the patient. A probable drug-related adverse event is assumed if the score is between 5 and 8, and a possible drug-related adverse event is assumed when the score is between 1 and 4. If the score is 0, it indicates that the adverse event is not due to the drug in use, but other factors.

#### 3.6.5 Treatment Acceptability Questionnaire

The TAQ comprises 4 questions: acceptability, efficacy, side effects, and trust rank of the therapist. All the responses on scale ranges from 1 to 7, in acceptability question 1 represents very unacceptable and 7 represents very acceptable, in efficacy question 1 represents very ineffective and 7 represents very effective, similarly in side effects question 1 represent very unlikely and 7 represent very likely and in trust rank of the therapist question 1 represents very untrustworthy and 7 represents very untrustworthy.^[48]^

### 3.7 Safety assessment, severity, and intensity of adverse drug reactions

To ensure the patient safety zolpidem 10 mg orally, not formal safety assessment protocol will be used as previous study reported that zolpidem was not associated with undesirable sleep side effects such as daytime drowsiness, headache, or amnesia.^[41]^

In order to make the interpretation of AEs more effective and in line with the evidence-based literature, *Naranjo algorithm* is known to be a valid measure for reporting and authenticating drug-related events.^[46,47]^ The adverse event will be recorded for zolpidem 10 mg oral tablets and if there is a major adverse event therapy should be considered for suspension. All the patients completing 6 weeks of zolpidem therapy will be questioned about any AEs that they may have experienced after taking zolpidem. The information will be collected based on the patients experience and the list of the AEs mentioned in Table 2.

**Table 2:**
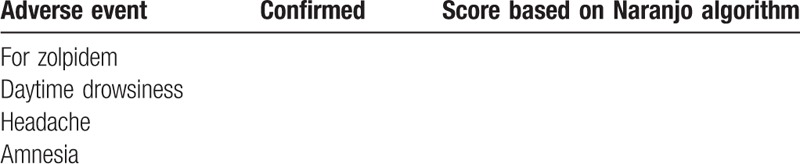
Assessment for confirm adverse events.

### 3.8 Dose titration

No titration will be done for zolpidem as we want to see the effect of zolpidem 10 mg (oral tablets) once daily.

### 3.9 Benefits for the patients

All study subjects participating in the study will undergo assessment for pruritus and sleep quality disturbing quality of life. Potentially of finding a better intervention among the patient, the patients would have beneficial effects for other patients elsewhere. Participating in the study patients would help in giving future directions to clinical practice in managing sleep disturbance and quality of life among CKD-associated pruritus patients undergoing hemodialysis.

### 3.10 Sample size

The sample size for the current study will be based on a statistical superiority trial (continuous data) design of an RCT. The hypothesis for this trial is that acupressure in intervention group is a more effective therapy to improve sleep quality among CKD-associated pruritus patients. The hypothesis testing is as follows: 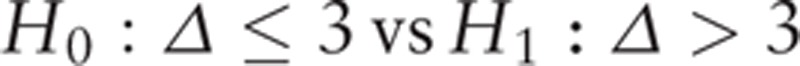


where 
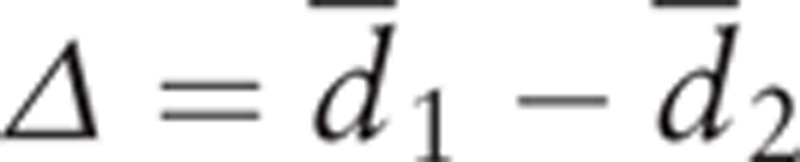
 is the difference of the mean differences of PSQI score before and after treatment between acupressure (
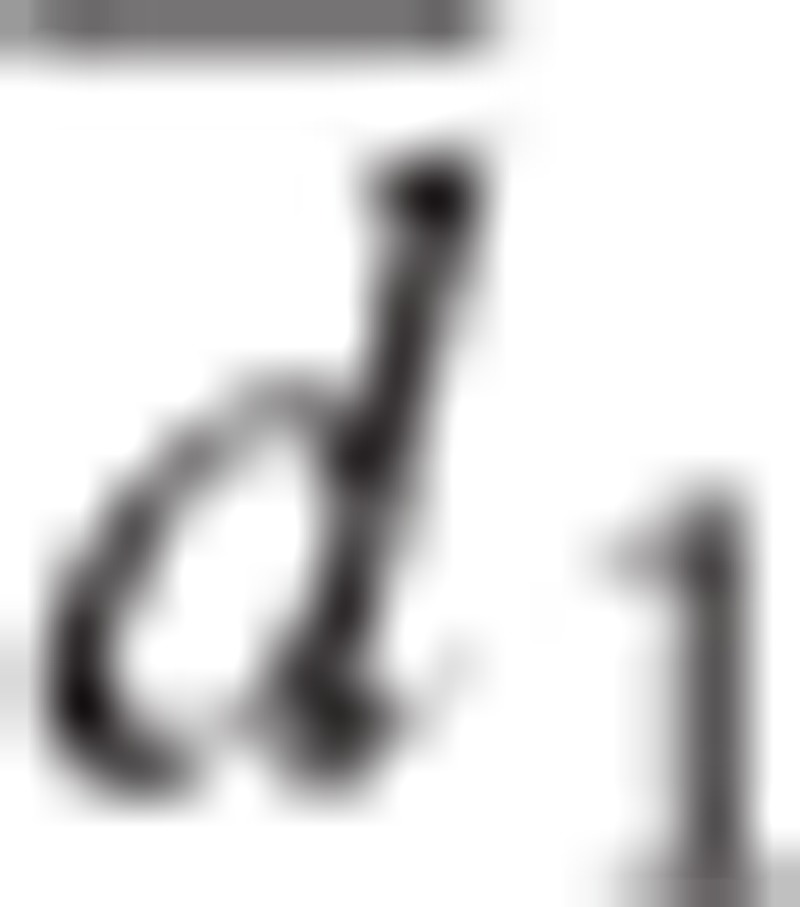
) and zolpidem group, that is, acupressure control (
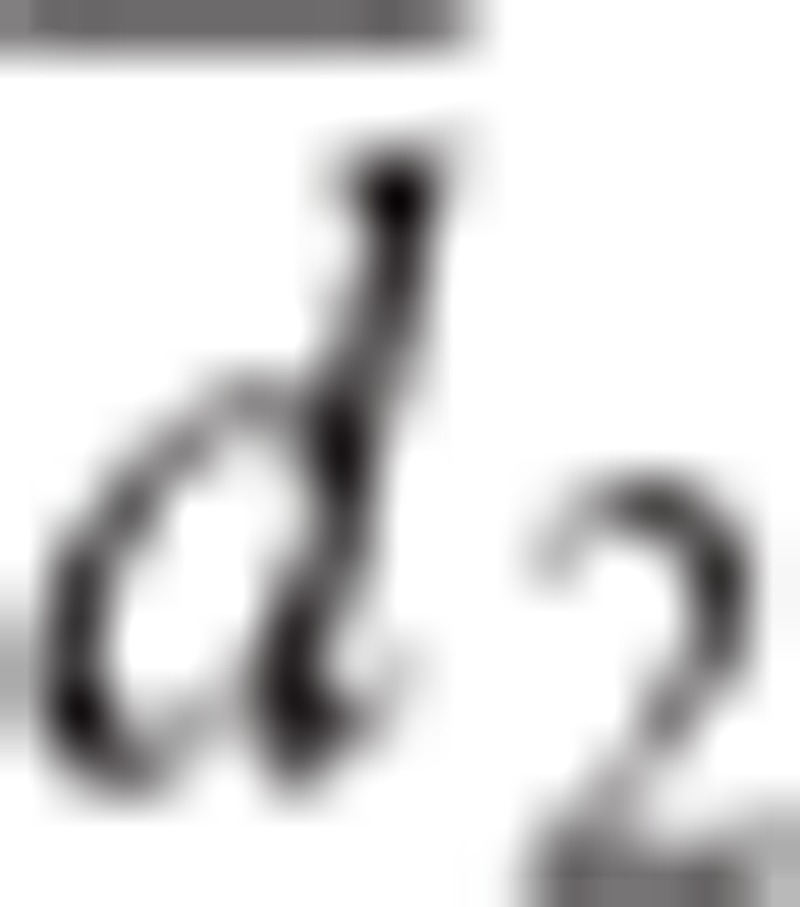
).

The sample size calculation was based on independent 2-sample test as patients from 2 parallel groups (acupressure vs zolpidem) can be considered independent samples as follows: 
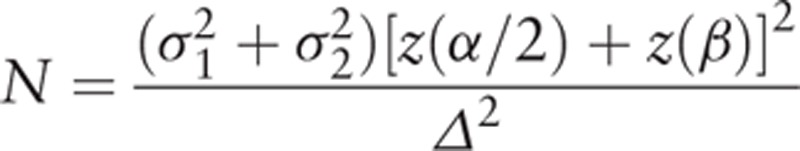


where *z*(α) and *z*(β) represent percentage points of the normal distribution for statistical significance level and power with α = 0.05 and β = 0.1, respectively;


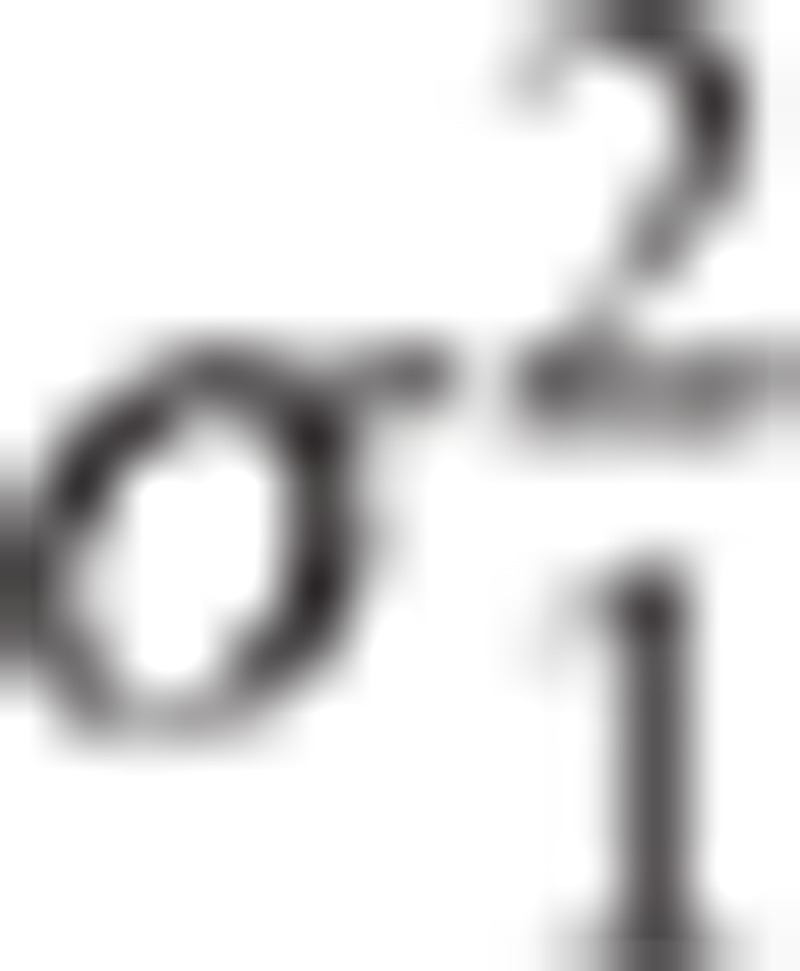
 and 
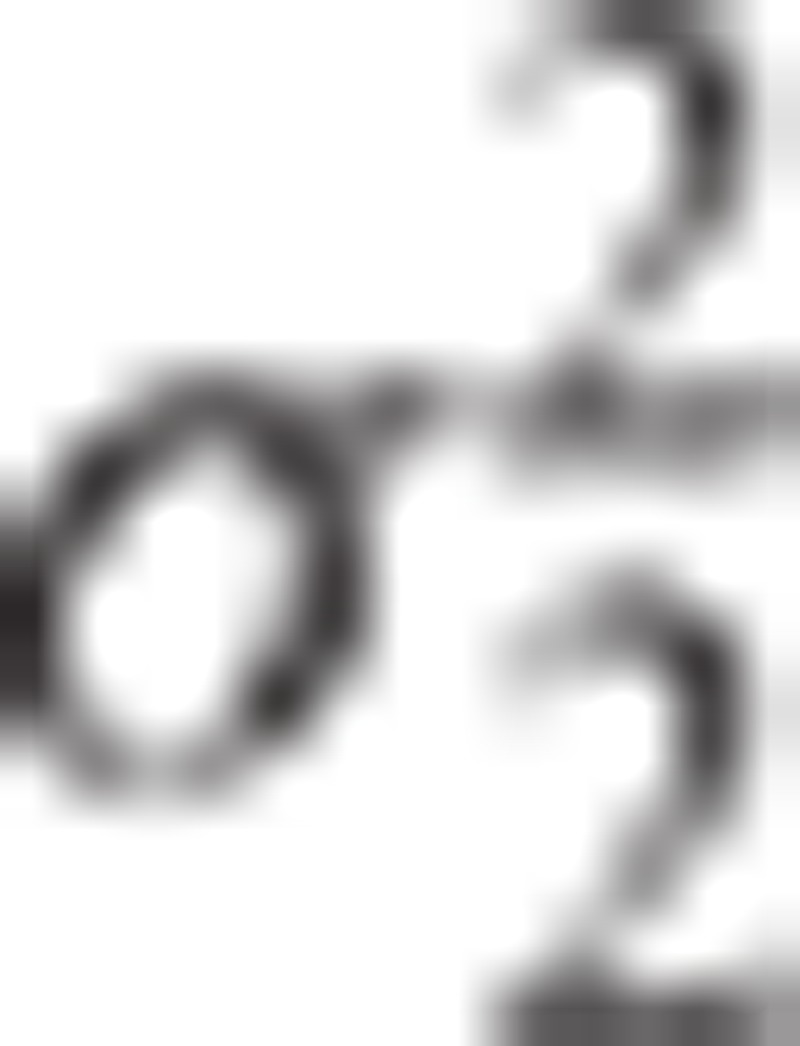
 were estimated based on a previously published study. Specifically, for example, for acupressure group, 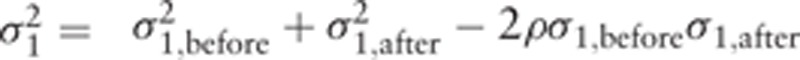 where the correlation (*ρ*) between before and after treatment was assumed to be 0.5.

Altogether 58 patients will be recruited for current RCT while considering the 20% drop out rate with 29 patients in each arm, that is, intervention group (zolpidem 10 mg) and acupressure group. The calculation was performed using STATA software version 15.0.

### 3.11 Statistical analysis

Statistical analysis will be performed by using SPSS 22. Baseline characteristics expressed as frequencies (percentages) for categorical variables, mean ± SD for continuous normally distributed variables, and median (interquartile range) for continuous non-normally distributed variables. PSQI global scores will be analyzed using analysis of covariance to examine the differences in treatment effects between the intervention and control groups.

## 4 Discussion

The management of pruritus-associated sleep in hemodialysis patients is often neglected by nephrologist. CKD-associated pruritus has major impact on quality of life. Pharmacological intervention with zolpidem 10 mg oral tablet is best choice in dialysis units for improving sleep.^[38–40]^ Nonpharmacological management such as acupressure therapy used in other previous clinical trials established to be the acceptable, safe, and well tolerated therapy for the management of sleep in patients with CKD on hemodialysis. Acupressure can be used in CKD-associated pruritus patients having sleep disorder while receiving hemodialysis therapy.

In study by Dashti-Khavidaki et al^[41]^ among hemodialysis patients by using zolpidem versus clonazepam; zolpidem was not associated with undesirable sleep side effects such as daytime drowsiness, headache, or amnesia, whereas clonazepam has been associated with severe side effects. About effectiveness of acupressure among hemodialysis patients Tsay et al,^[49]^ reported an improvement in sleep quality of dialysis patients as result of acupressure on the Shenmen points of the wrists. A study by Nasiri et al,^[50]^ also support an improvement in sleep quality in hemodialysis patients by acupressure in both the control and intervention group. Findings of Arab et al^[51]^ suggesting that acupressure result in a short-term improvement in sleep quality of hemodialysis patients and also bring comfort to patients and enhance their quality of life.

Overall, previous study of acupressure on sleep quality among hemodialysis patients showed positive impact on improving the sleep quality. Clinicians should consider providing acupressure as an alternative method to improving dialysis patients’ quality of sleep. Nurses, patients, and their families could be easily trained to administer acupressure to those who have sleep disturbance.

### 4.1 Strengths and limitations

This interventional study is first of its kind to be conducted involving randomized controlled design. The finding of better intervention among the patient, the results would have beneficial effects for other patients elsewhere. Participating in the study patients would help in giving future directions to clinical practice in managing sleep disturbance and quality of life among CKD-associated pruritus patients undergoing hemodialysis. A possible limitation of the study is drop out, To reduce the chances of drop-out, an additional 20% sample population will be recruited to compensate the drop-out.

## Author contributions

**Conceptualization:** Inayat Ur Rehman, Tahir Mehmood Khan.

**Data curation:** Inayat Ur Rehman, Raheel Ahmed, Nisar Ahmad Khan, Aziz Ur Rehman, Syed Munib.

**Formal analysis:** Inayat Ur Rehman, David wu bin Chia, Tahir Mehmood Khan.

**Investigation:** Inayat Ur Rehman, Tahir Mehmood Khan, Lee Learn Han, Kok Gan Chan.

**Methodology:** Inayat Ur Rehman, Tahir Mehmood Khan.

**Project administration:** Inayat Ur Rehman, Tahir Mehmood Khan.

**Supervision:** Inayat Ur Rehman, Tahir Mehmood Khan.

**Validation:** Inayat Ur Rehman, Tahir Mehmood Khan.

**Visualization:** Inayat Ur Rehman.

**Writing – original draft:** Inayat Ur Rehman.

**Writing – review and editing:** Tahir Mehmood Khan, Lee Learn Han.
