# A randomized controlled trial for effectiveness of zolpidem versus acupressure on sleep in hemodialysis patients having chronic kidney disease–associated pruritus: Erratum

**DOI:** 10.1097/MD.0000000000012527

**Published:** 2018-09-14

In the article, “A randomized controlled trial for effectiveness of zolpidem versus acupressure on sleep in hemodialysis patients having chronic kidney disease–associated pruritus”,^[1]^ which appeared in Volume 97, Issue 31 of *Medicine*, Dr. David Bin-Chia Wu's name appeared incorrectly as David Wu Bin Chia.
